# Clustering and Machine Learning Models of Skeletal Class I and II Parameters of Arab Orthodontic Patients

**DOI:** 10.3390/jcm14030792

**Published:** 2025-01-25

**Authors:** Kareem Midlej, Osayd Zohud, Iqbal M. Lone, Obaida Awadi, Samir Masarwa, Eva Paddenberg-Schubert, Sebastian Krohn, Christian Kirschneck, Peter Proff, Nezar Watted, Fuad A. Iraqi

**Affiliations:** 1 Department of Clinical Microbiology and Immunology, Faculty of Medicine and Health Sciences, Tel Aviv University, Tel Aviv 6997801, Israel; kareemmidlej@mail.tau.ac.il (K.M.); osaydzohud@mail.tau.ac.il (O.Z.); iqbalzoo84@gmail.com (I.M.L.); 2 Center for Dentistry Research and Aesthetics, Jatt 4491800, Israel; awadi.obaida@gmail.com (O.A.); sameer.massarwa@gmail.com (S.M.); nezar.watted@gmx.net (N.W.); 3 Department of Orthodontics, University Hospital of Regensburg, University of Regensburg, 93047 Regensburg, Germany; eva.paddenberg@klinik.uni-regensburg.de (E.P.-S.); sebastian.krohn@klinik.uni-regensburg.de (S.K.); peter.proff@klinik.uni-regensburg.de (P.P.); 4 Department of Orthodontics, University of Bonn, 53111 Bonn, Germany; christian.kirschneck@uni-bonn.de; 5 Department of Orthodontics, Faculty of Dentistry, Arab America University, Jenin 919000, Palestine; 6 Gathering for Prosperity Initiative, Jatt 4491800, Israel

**Keywords:** skeletal malocclusion, skeletal deformities, cephalometric parameters, disease classification, machine learning

## Abstract

**Background**: Orthodontic problems can affect vital quality of life functions, such as swallowing, speech sound production, and the aesthetic effect. Therefore, it is important to diagnose and treat these patients precisely. The main aim of this study is to introduce new classification methods for skeletal class I occlusion (SCIO) and skeletal class II malocclusion (SCIIMO) among Arab patients in Israel. We conducted hierarchical clustering to detect critical trends within malocclusion classes and applied machine learning (ML) models to predict classification outcomes. **Methods**: This study is based on assessing the lateral cephalometric parameters from the Center for Dentistry Research and Aesthetics based in Jatt, Israel. The study involved the encoded records of 394 Arab patients with diagnoses of SCIO/SCIIMO, according to the individualized ANB of Panagiotidis and Witt. After clustering analysis, an ML model was established by evaluating the performance of different models. **Results**: The clustering analysis identified three distinct clusters for each skeletal class (SCIO and SCIIMO). Among SCIO clusters, the results showed that in the second cluster, retrognathism of the mandible was less severe, as represented by a lower ANB angle. In addition, the third cluster had a lower NL-ML angle, gonial angle, SN-Ba angle, and lower ML-NSL angle compared to clusters 1 and 2. Among SCIIMO clusters, the results also showed that the second cluster has less severe retrognathism of the mandible, which is represented by a lower ANB angle and Calculated_ANB and a higher SNB angle (*p* < 0.05). The general ML model that included all measurements for patient classification showed a classification accuracy of 0.87 in the Random Forest and the Classification and Regression Tree models. Using ANB angle and Wits appraisal only in the ML, an accuracy of 0.78 (sensitivity = 0.80, specificity = 0.76) was achieved to classify patients as SCIO or SCIIMO. **Conclusions:** The clustering analysis revealed distinguished patterns that can be present within SCIO and SCIIMO patients, which can affect the diagnosis and treatment plan. In addition, the ML model can accurately diagnose SCIO/SCIIMO patients, which should improve precise diagnostics.

## 1. Introduction

Angle classification is one of the most widely used methods in malocclusion classifications [1,2]. Angle class I molar classification is determined by the mesiobuccal cusp of the maxillary first molar occluding with the buccal groove of the mandibular first molar [3]. Class I malocclusion was also categorized into five types by Dewey’s modification. In Dewey type 1, anterior teeth are crowded, and in Dewey type 2, maxillary incisors are proclined. In type 3, there is an anterior crossbite, whereas in type 4, there is a posterior crossbite. Type 5 is characterized by permanent molars’ mesial drifts [3,4]. Skeletal class II malocclusion (SCIIMO)’s etiology is heterogeneous because it can be caused by a mandibular retrusion, maxillary protrusion, or both [1,5]. Also, in SCIIMO, patients are categorized by the interarch relation. In a Class II molar relationship, the mesiobuccally cusp of the maxillary first permanent molar occludes mesial to the buccal groove of the mandibular first molar with correct inclination of the front teeth. Class II division 1 occurs when the maxillary incisors are protruded (upper incisors are proclined), often resulting in an excessive overjet and deep overbite. Class II division 2 occurs when the maxillary central incisors are palatally inclined and eventually overlapped by the maxillary lateral incisors. A deep overbite and a broad maxillary arch define a class II division 2 [3].

Many approaches have been applied to diagnose craniofacial anatomy, commonly based on bidimensional imaging [6]. In recent years, three-dimensional (3D) technologies have been developed, and more tools have been enabled in orthodontics [4].

3D technologies can define the occurrence of malocclusion: sagittal, transverse, and vertical [7,8]. In the sagittal plane, the intermaxillary angle (SNA − SNB = ANB), which was recommended by Steiner 5 to determine an individual’s skeletal class, indicates skeletal class I occlusion (SCIO) if the ANB angle has values ranging between 0° and 4°, and SCIIMO if the ANB angle presents values > 4° [9,10]. According to Jacobson [11], the ANB angle does not consider the relative relationship of the denture bases to cranial reference planes. Due to this limitation, the “Wits” appraisal parameter was suggested, which overcomes this shortcoming and enables the measurement of the severity of the degree of anteroposterior jaw disharmony from lateral cephalograms [11,12]. Over subsequent years, numerous research studies have provided formulas that take into account the distinct characteristics of the ANB angle and Wits evaluation [13,14,15,16,17].

In summary, all traditional skeletal classifications depend on manually calculating linear and angular variables using the craniomaxillary and mandibular landmarks. However, skeletal classification can be difficult due to variations in the mandible’s position that can result from occlusion and temporomandibular joint [18]. To overcome these difficulties, classification algorithms like the Support Vector Machine (SVM) can generate skeletal classifications based on the automatically extracted craniomaxillary variables [18,19]. Another study that examined automated skeletal classification found that convolutional neural networks (CNNs), using the patient’s sex and a cephalogram, exhibited > 90% sensitivity, specificity, and accuracy for vertical and sagittal skeletal diagnosis [20]. Taraji et al. [21] used cephalometric parameters, along with covariates such as gender, age, and race, to appraise machine learning algorithms for adult Class III malocclusion treatment planning. Their study demonstrated that artificial neural network algorithms predicted treatment approach with 91% accuracy. The model highlighted Wits appraisal, anterior overjet, and Mx/Md ratio as key predictors.

A scoping review that evaluated the use of artificial intelligence in orthodontics and included 62 studies demonstrated that most of the studies originated from the USA, South Korea, Japan, and China. In addition, the review revealed that the diagnosis and treatment planning field was one of the major domains that were investigated [22].

Therefore, the main aim of this study was to derive a novel classification machine learning (ML) model to predict whether it is SCIO or SCIIMO malocclusion among Palestinian Arab residents of Israel, using lateral cephalogram parameters, in addition to gender and age as covariates. This population can be considered a permanent population of this area, with family histories dating back for generations and high levels of consanguinity.

To our knowledge, this research will be the first to apply machine learning models to this ethnic group. As a secondary outcome, a clustering analysis was intended to represent the data in clusters and to examine the differences between these clusters.

## 2. Material and Methods

### 2.1. Ethical Statement

This research was conducted according to current guidelines of the ethics and regulations of the University of Regensburg Ethics Committee (approval number 19-1596-101 (dated 13 November 2019)). This study consisted of 394 coded records of Palestinian Arab citizens of Israel who were diagnosed as SCIO or SCIIMO. All information was collected as part of the standard of care by the orthodontists’ team at the Center for Dentistry Research and Aesthetics based in Jatt, Israel.

The inclusion criteria were:

1. Patients diagnosed with SCIO (−1 ≤ Calculated_ANB ≤ 1) or SCIIMO (Calculated_ANB > 1).

Calculated_ANB is defined as equal to ANB measured − ANB_ind_. The ANB_ind_ was defined by Panagiotidis and Witt [9]: ANB_ind_ = −35.16 + (0.4 × SNA) + (0.2 × ML-NSL).

In some cases, patients were included as SCIO or SCIIMO, even when the Calculated_ANB was not in the accepted range, medical diagnosis, and additional important variables, like the ANB angle and Wits appraisal. The reason that the Calculated_ANB is not suitable for all patients was described by Panagiotidis and Witt [13].

2. Available pre-treatment cephalometric data.

The exclusion criteria were:

1. Participants/parents/guardians who could not sign an informed consent form.
2. Orthodontic patients who had already started the treatment process.

In this study, we performed a clustering analysis. In addition, we applied various ML algorithms, which differed in the number of input variables, enabling us to precisely classify the patients as skeletal class I or II via automatic machine learning techniques.

### 2.2. Cephalometric Variables

The following cephalometric parameters were the essential variables in this study analysis, and the complete information and location of all the parameters are presented in Supplementary Table S1 and Supplementary Figure S1.

### 2.3. Descriptive Data Analysis

Data was analyzed using the R software (R-4.3.1) platform. Descriptive statistics performed to both SCIO and SCIIMO patients and included the covariates gender and age. In addition, descriptive statistics were performed on all cephalometric parameters and included sample size, mean, standard deviation, minimum value, 25th percentile, 75th percentile, and maximum value.

### 2.4. Clustering Analysis

Data was analyzed using the R software platform, Clustering Analysis. Before starting the clustering process, we conducted scaling to reach a common scale.

The clustering algorithm was performed for 3 clusters, including SCIIMO or SCIO patients (separately). A scatter plot and dendrogram were produced using the R statistical program (R-4.3.1) to implement the visualization of the cluster analysis results. We got the optimal cluster number by inspecting the dendrogram of different clusters.

To implement a hierarchical clustering algorithm, one has to choose a linkage function that defines the distance between any two subsets [23]. In all our clustering calculations, we mainly used the Ward error sum of squares hierarchical clustering method that has been commonly used since its first description by Ward in 1963 [24].

The performance of the machine learning models was evaluated by determining the accuracy and kappa scores.

### 2.5. Machine Learning Methods

Machine learning analysis was applied using the R package Caret (R-4.3.1) [25]. Before starting this analysis, we preprocessed the data with centering and scaling functions to reach a standard scale and to improve the performance of the models.

### 2.6. Classification Techniques

The classification techniques employed included Linear Discriminant Analysis (LDA), Support Vector Machine (SVM), K-Nearest Neighbor (KNN), Random Forest (RF), and Classification and Regression Tree (CART). All methods were implemented with K-fold cross-validation, where K was set to 10.

### 2.7. Linear Discriminant Analysis (LDA)

LDA was proposed by R. Fischer in 1936. It consists of finding the projection hyperplane that minimizes the interclass variance and maximizes the distance between the projected means of the classes [26].

### 2.8. Support Vector Machine (SVM)

The machine conceptually implements the idea that input vectors are non-linearly mapped to a high-dimension feature space. In this feature space, a linear decision surface is constructed [27]. This model can be relatively simple and flexible for addressing various classification problems. SVMs afford balanced predictive performance distinctively, even in studies with limited sample sizes [28].

### 2.9. K-Nearest Neighbor (KNN)

The nearest neighbor decision rule assigns an unclassified sample point to the classification of the nearest of a set of previously classified points. The k defines how many nearest neighbors need to be examined to classify the class of a sample point [29,30,31]. This investigation utilized accuracy as the criterion for selecting the optimal model, prioritizing the one with the highest k-value. The final value was applied to develop the general model (all parameters), model 1 (ANB angle), and model 2 (ANB angle and Wits appraisal), was 9 (k = 9), while the model without the ANB parameters was k = 5 (5 neighbors).

### 2.10. Random Forest (RF)

Random Forest is a classification method that uses many decision trees. This algorithm is a combination of tree predictors such that each tree depends on the values of a random vector sampled independently and with the same distribution for all trees in the forest [31,32].

### 2.11. Classification and Regression Tree (CART)

CART analysis is a form of binary recursive partitioning. In this method, each group of patients, represented by a “node” in a decision tree, can only be split into two groups. Each parent node can be split into two child nodes. The term “partitioning” refers to the fact that the dataset is split into sections or partitioned. It is important to know that CART can handle numerical data that are highly skewed or multi-modal, as well as categorical [33].

### 2.12. Model Validation

We divided our data into 70% for training and 30% of the data for validation (unseen data). Our models were validated using the k-fold cross-validation technique, a straightforward and efficient approach for model selection and performance evaluation. In k-fold cross-validation, the dataset is randomly divided into *k* disjoint subsets of roughly equal size [34,35]. This research employed 10-fold cross-validation. For conducting the ML models and calculating the accuracy, kappa, receiver–operating characteristic curve (ROC), sensitivity, and specificity scores, we used the R package Caret [25].

### 2.13. Variables Contribution to the Classification of SCIO and SCIIMO

Finally, we used the variable importance using the function available in the R Caret package (R version used-4.3.1) [21] to summarize the importance of each parameter to the model in predicting the classification of skeletal classes I and II.

## 3. Results

This study included 394 patients, with 157 (39.8%) presenting SCIO and 237 (60.2%) presenting SCIIMO. The total study collective consisted of patients aged between 7 and 55 years, with a mean age of 19 ± 7.1 years in class I and 17 ± 6.5 years in class II cases. Concerning the gender distribution, among SCIO patients, 66% were female (n = 104), and 67% were female (n = 158) in class II. The characteristics of the study collective, including demographic and cephalometric data, are presented in Table 1 and Table 2 for SCIO and SCIIMO patients, respectively.

### 3.1. Clustering

In this section, we performed hierarchical clustering analysis and inspected the number of clusters according to the dendrogram result. It was acceptable to present the current results with k = 2, 3, and 4 clusters. However, we decided to describe the k = 3 clusters and show the distinct variations between the clusters. The same analysis was performed for skeletal class I and II separately.

### 3.2. Skeletal Class I Occlusion (SCIO) Clustering

**Figure 1:**
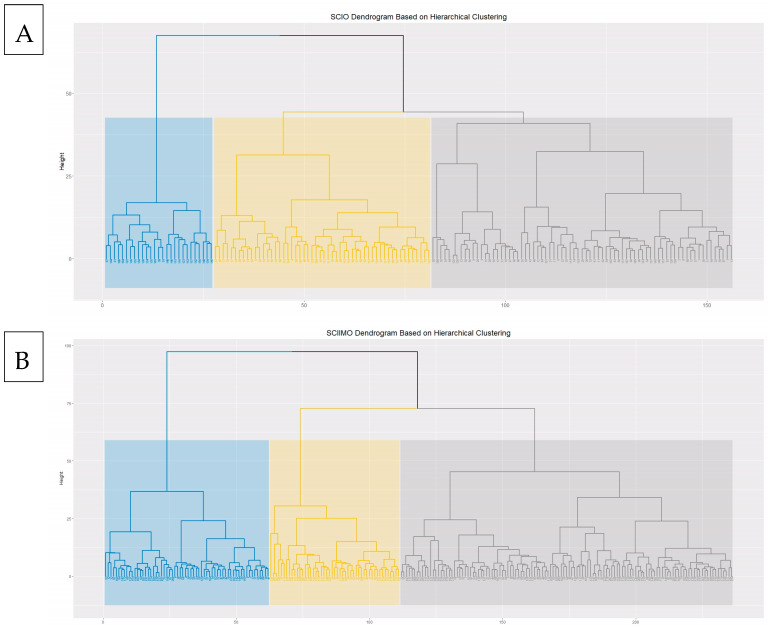
(**A**) Hierarchical clustering dendrogram for skeletal class I occlusion (SCIO). *x*-axis (rows) represents patient clustering, divided into three main clusters with different colors, and *y*-axis represents distance between clusters. (**B**) Hierarchical clustering dendrogram for skeletal class II malocclusion (SCIIMO). *x*-axis (rows) represents patients clustering, divided into three main clusters with different colors, and *y*-axis represents distance between clusters.

The hierarchical clustering results showed that the majority of patients were assigned to cluster 1 consisted of 75 (n = 75) patients, and cluster 2 (n = 54), while 27 patients (n = 27) were assigned to the third cluster (Figure 1A and Table 3).

In addition, the three clusters interestingly variated significantly (*p* < 0.05) in all cephalometric parameters except in the parameters (Calculated_ANB and Wits appraisal, *p* > 0.05). In addition, the results showed that age differences were not significant between the three clusters. The results showed that in the second cluster, retrognathism of the mandible was less severe, as represented by a lower ANB angle. In addition, the third cluster had a lower NL-ML angle, gonial angle, SN-Ba angle, and lower ML-NSL angle compared to clusters 1 and 2, demonstrating the distinct features from the other two clusters. Detailed information can be found in Table 4.

We repeated the same clustering analysis with males and females separately and found that there are some similar patterns, like the fact that in both males and females, the second cluster was characterized by less severe retrognathism of the mandible, as represented by a lower ANB angle. However, the results also showed variations between males and females. For instance, most dental parameter variations were not significant between the three clusters among males, and were significant among females clustering. The results showed that age was insignificant between clusters for both males and females. Overall, females showed more significant differences among clusters among the cephalometric parameters (Table 5).

### 3.3. Skeletal Class II Malocclusion (SCIIMO) Clustering

In skeletal class II hierarchical clustering, the results showed that the majority of patients were assigned to cluster 1, consisting of 125 (n = 125) patients. In contrast, 111 patients (cluster 2, n = 62, cluster 3, n = 49) were assigned the second and third clusters (Figure 1B and Table 3).

In addition, the three clusters interestingly variated significantly (*p* < 0.05). Also, among skeletal class II patients, age differences in the different clusters were not significant. Interestingly, the results showed a significant difference among most of the sagittal parameters, especially the ANB angle and the Calculated_ANB. The results showed that the second cluster has less severe retrognathism of the mandible, which is represented by a lower ANB angle and Calculated_ANB and a higher SNB angle (*p* < 0.05). Detailed information can be found in Table 6.

Finally, we repeated the same clustering analysis with males and females separately and found that age differences were significant between clusters for males and females (*p* < 0.05). Cluster 1 here was characterized by a younger age average M = 12.90 (M = 12.90, SD = 2.20) among males and M = 15.88 (M = 15.88, SD = 5.11). For both males and females, the results showed that the first cluster has less severe retrognathism of the mandible, which is represented by a lower ANB angle and Calculated_ANB (significant among males only, *p* < 0.05), compared to the other two clusters. In addition, and contrary to skeletal class I, here, Wits appraisal variations were significant between the clusters for both males and females (*p* < 0.05) and were lower in the first cluster compared to the other two clusters. Overall, also among skeletal class II patients, females showed more significant differences among clusters among the cephalometric parameters (Table 7).

### 3.4. Machine Learning Models

Considering the knowledge about cephalometric measurements in SCIO and SCIIMO obtained from cluster analysis and comparisons of cephalometric parameters between subgroups, various machine learning models were tested to predict the classification of an individual based on machine learning (ML) models that will not be based on the Calculated_ANB (model 3).

**Figure 2:**
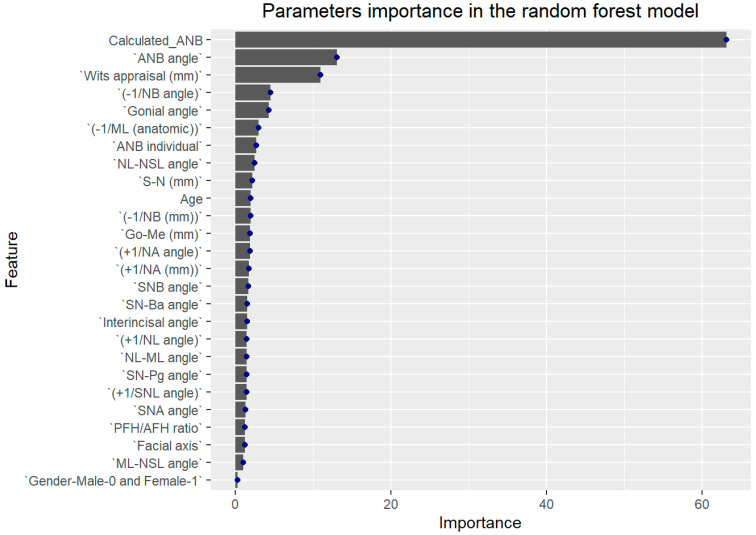
General machine learning model summary of importance of each parameter to model in predicting classification of skeletal class I or II. X-axis shows importance of prediction of different assessed variables. Y-axis shows list of the assessed variables. In this figure, we arranged parameters according to their contribution to the model. In this figure, we can see that Calculated_ANB was most important and contributed more than any other parameter.

The general ML model, which included all cephalometric and demographic parameters, could predict a SCIO or SCIIMO with an accuracy of 0.87 (0.87%) (RF, Accuracy = 0.87, Kappa = 0.74, ROC = 0.92, Sensitivity = 0.92, and Specificity = 0.83). As expected, Calculated_ANB was the most critical parameter in the model, followed by Wits appraisal, ANB, −1/NB, and gonial angle, as shown in Figure 2.

**Figure 3:**
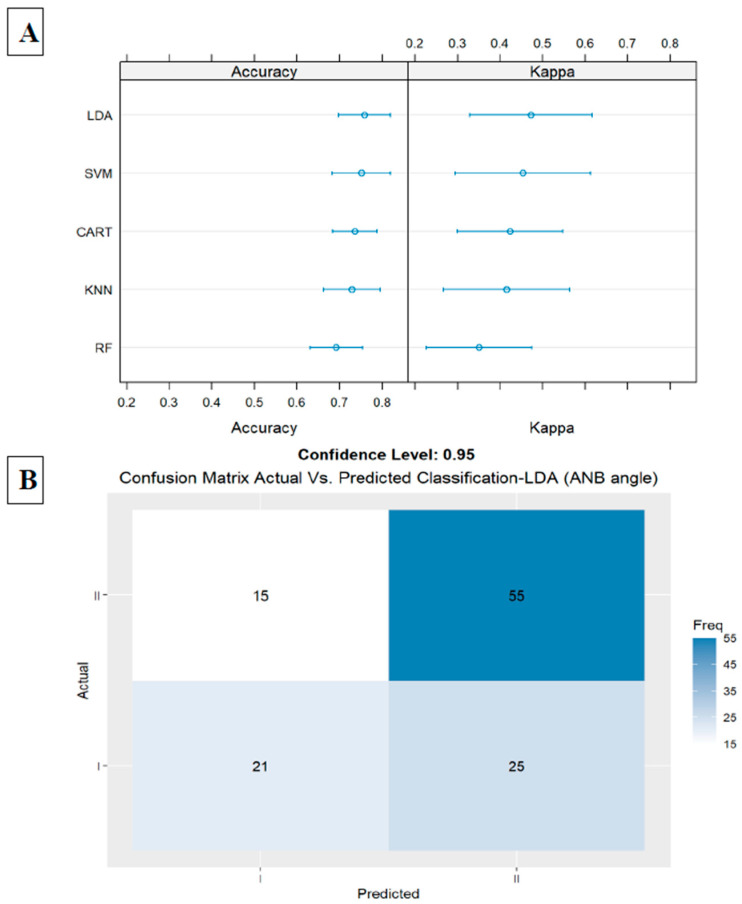
(**A**) Summarize model 1 (one predictor) of the different machine learning models. This figure presents a summary of the five Machine Learning classification models, including Linear Discriminant Analysis (LDA), Support Vector Machine (SVM), K-Nearest Neighbor, Random Forest (RF), Classification, and Regression Tree (CART), presented in Y-axis. X-axis shows accuracy and kappa scores for each model. First model includes ANB angle only; in LDA, we received an accuracy of 0.75 and kappa of 0.47. (**B**) Summarize model 1 (one predictor) of the different machine learning models. Presents LDA Machine Learning Model Confusion Matrix of validation data (30% of the sample) for ANB angle to predict classification (Predicted) compared to actual classification, based on using this variable only. X-axis shows skeletal class I and II predictions, and Y-axis shows number of identified patients in each classification.

The first model included only the ANB angle, the second important parameter in the general model. In this model, we received an accuracy of 0.75 (LDA, accuracy = 0.75, kappa = 0.47, ROC = 0.79, sensitivity = 0.59, and specificity = 0.86). Results are presented in Figure 3A,B.

**Figure 4:**
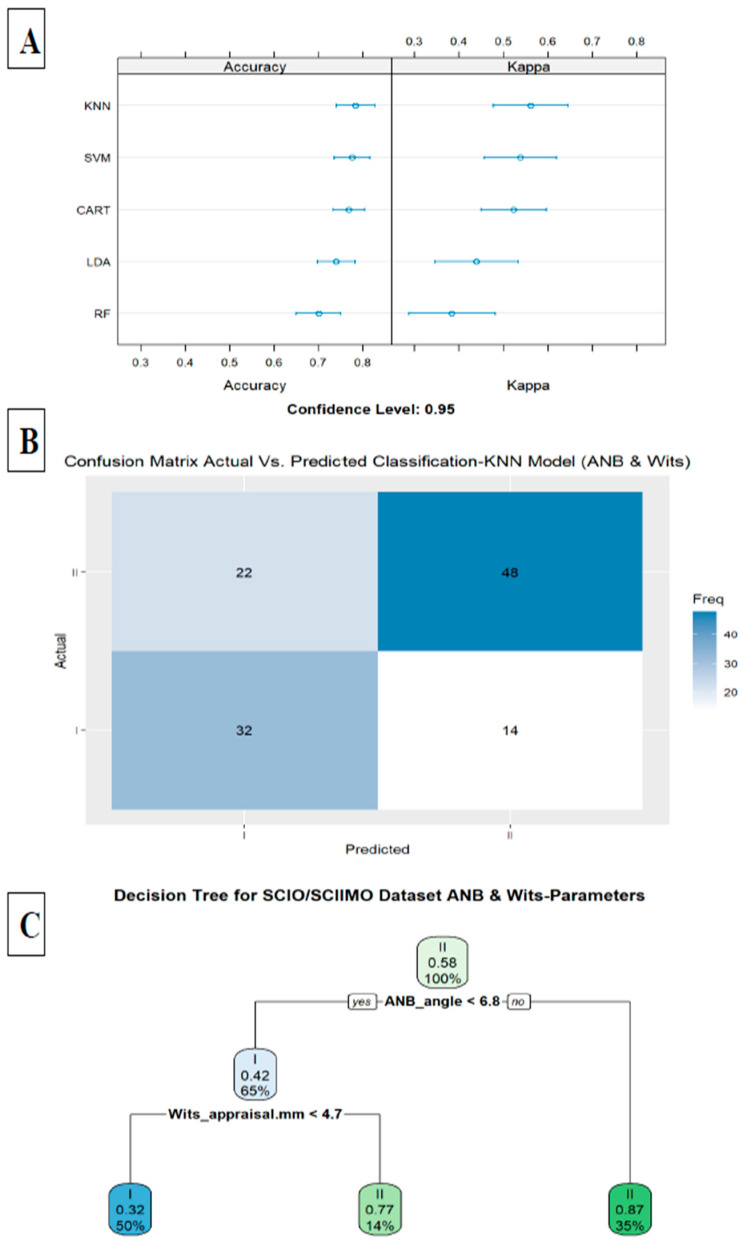
(**A**) Summarize model 2 (two predictors) of different machine learning models. This figure presents a summary of five machine learning classification models tested, including Linear Discriminant Analysis (LDA), Support Vector Machine (SVM), K-Nearest Neighbor, Random Forest (RF), Classification and Regression Tree (CART) as presented on Y-axis. X-axis shows accuracy and kappa scores for each model. (**B**) Machine Learning Model Confusion Matrix of validation data shows the ability of KNN model to predict classification (Predicted) compared to Actual classification based on ANB angle and Wits appraisal. X-axis shows skeletal class I and II predictions, and Y-axis indicates number of identified patients in each classification. (**C**) Second model tree diagram shows decision rules of model. Root node is at top, and leaf nodes are at bottom. Each node is labeled with the cephalometric parameter used to split the data at that node, as well as the split value. Leaf nodes are labeled with predicted class for data that reaches that node.

**Figure 5:**
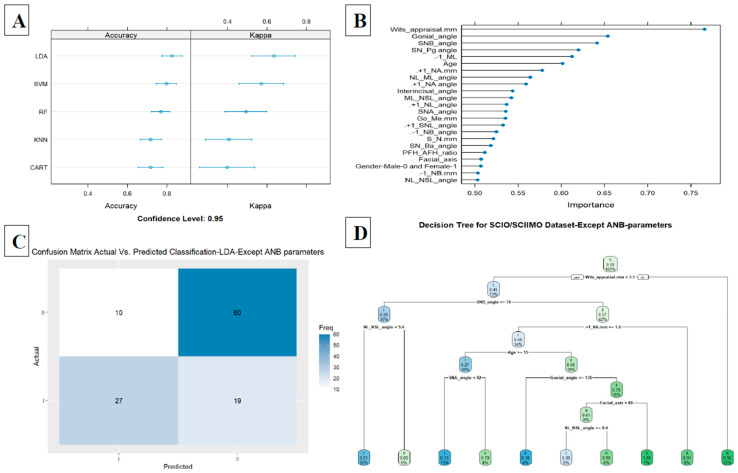
(**A**) Summarize model 3 (all parameters except ANB angle, ANBind, and Calculated_ANB) of different machine learning models. This figure presents a summary of five machine learning classification models tested, including Linear Discriminant Analysis (LDA), Support Vector Machine (SVM), K-Nearest Neighbor, Random Forest (RF), Classification and Regression Tree (CART) as presented on Y-axis. X-axis shows accuracy and kappa scores for each model. (**B**) Model 3 summarizes importance of each parameter to the model in predicting classification of skeletal classes I and II. X-axis shows the importance of prediction for different assessed variables. Y-axis shows list of assessed variables. (**C**) Machine Learning Model Confusion Matrix of validation data. It shows ability of LDA model to predict classification (Predicted) compared to Actual classification based on individualized ANB angle (Calculated_ANB). X-axis shows skeletal class I and II predictions, and Y-axis indicates number of identified patients in each classification. (**D**) Second model tree diagram shows decision rules of model. Root node is at top, and leaf nodes are at bottom. Each node is labeled with cephalometric parameter used to split the data at that node and the split value. Leaf nodes are labeled with predicted class for data that reaches that node.

The second model included the ANB angle and the Wits appraisal, which improved the accuracy to 0.78 in the KNN model (KNN, accuracy = 0.78, kappa = 0.56, ROC = 0.82, sensitivity = 0.80, and specificity = 0.76) (Figure 4A,B). A decision tree visualization for these two parameters is presented in Figure 4C. Finally, adding the third model (model 3) included all parameters except for ANB, ANB_ind_, and Calculated_ANB, and achieved 0.82 accuracy in the LDA model (LDA, accuracy = 0.82, kappa = 0.63, ROC = 0.88, sensitivity = 0.75, and specificity = 0.87). In addition, we examined the classification ability without ANB, ANB_ind_, and Calculated_ANB parameters via decision tree, which showed that the tree starts with Wits appraisal, followed by SNB, NL-NSL, +1/NA (mm), and age in the first three branches (Figure 5A–D). A summary of all the ML models is available in Table 8.

## 4. Discussion

Our study aimed to reveal novel information about the Palestinian Arab ethnic minority who are citizens of Israel. Hierarchical clustering analysis was performed separately for both skeletal class I and II patients and based on the dendrogram we decided to apply three clusters for every analysis. Among skeletal class I patients, three distinct patterns were revealed. The second cluster was characterized by less severe retrognathism. For skeletal class II patients, we also applied hierarchical clustering for three clusters, and here also the results showed a significant among most of the sagittal parameters, especially the ANB angle and the Calculated_ANB. The results showed that the second cluster has less severe retrognathism of the mandible, which is represented by a lower ANB angle and Calculated_ANB and a higher SNB angle. Interestingly, age differences were significant between clusters among males and females.

In a study by Moreno Uribe et al. [36] about phenotypic diversity in white adults with Class II malocclusion, it was found that models with two, three, or four clusters were statistically acceptable. Still, they identified five distinct Class II phenotypes [36].

A Cluster analysis study in Class I occlusion revealed that the grouping pattern in Class I occlusion is present at younger age levels and disappears with age. Also, they found that the clustering pattern is very similar in males and females with Class I occlusion [37].

Finally, the general ML model that included all parameters could predict an individual as SCIO or SCIIMO with 0.87 accuracy (RF, accuracy = 0.87, kappa = 0.74, ROC = 0.92, sensitivity = 0.92, and specificity = 0.83). As expected, Calculated_ANB was the most critical parameter in the model, followed by ANB angle, Wits appraisal, and ANB_ind_. Our machine learning results demonstrated that with the ANB angle and the Wits appraisal, two cephalometric parameters can predict skeletal class I/ II with 0.78 accuracy (KNN, accuracy = 0.78, kappa = 0.56, ROC = 0.82, sensitivity = 0.80, and specificity = 0.76). In addition, our third model (model 3) included all parameters except for ANB, ANB_ind_, and Calculated_ANB, and achieved 0.82 accuracy in the LDA model (LDA, accuracy = 0.82, kappa = 0.63, ROC = 0.88, sensitivity = 0.75, and specificity = 0.87).

Recent research that was conducted by Midlej et al. [38] aiming to accurately categorize Arab patients, identified as citizens of Israel, individually categorized as skeletal class II or III, found that Wits appraisal and SNB angle were capable of forecasting the classification of patients as either skeletal class II or III, achieving an accuracy of 0.95.

Jayathilake et al. [39] examined the prediction of malocclusion patterns using a classification model. Their study considered SNA, SNB, and ANB as cephalometric variables. The patients were classified into malocclusion patterns according to the ANB angle (pattern I, II, and III). The study revealed that the accuracy rates for predicting malocclusion patterns were as follows: 88.89% for the multinomial logistic regression model, 83.33% for the K-NN algorithm, 88.89% for Random Forest, and 55.56% for the Naïve Bayes classifier.

In another study that aimed to accurately diagnose skeletal class III malocclusion applied through mobile images, using three models (a deep learning algorithm, a machine learning algorithm, and a rule-based algorithm), found that the best model was able to correctly classify skeletal class III malocclusion, with an accuracy of 76% [40].

### 4.1. Traditional vs. New Machine Learning Methods

When comparing the performance of ML models compared to traditional diagnostic approaches, like the Calculated_ANB formula that we used in this article to classify the patients. We need to consider that this regression formula (Calculated_ANB) and others do not fit all cases. In many cases, patients are diagnosed clinically, and in accordance with additional cephalometric metrics, such as Wits assessment and ANB angle. Panagiotidis and Witt [13] investigated this limitation by the correlation coefficient of the ANB_ind_ equation, as r = 0.808. In addition, the available individualized were established based on specific populations, considering the individual properties of the ANB angle and Wits appraisal [13,14,15,16,17]. On the other hand, this research and other studies suggest applying uniform machine learning algorithms that can be trained on one ethnic group, like this study, or future studies that will aim to combine different ethnicity models.

### 4.2. Limitations

This study applied clustering analysis only on three clusters. Although the results showed significant patterns within each skeletal class, further investigations for different cluster numbers could be conducted. This study used a machine learning model based on a moderate sample size, and future studies should consider increasing it. Furthermore, our results were drawn from specific populations, and the results might vary among other populations. Finally, future research should aim to include all skeletal malocclusion classifications, not only skeletal classes I and II.

### 4.3. Conclusions and Future Research

This research revealed new information regarding the distinct characteristics of each cluster within each patient group (SCIO/SCIIMO) based on various cephalometric parameters.

Among skeletal class I patients, three distinct patterns were revealed. The second cluster was characterized by less severe retrognathism. Regarding skeletal class II patients, the three clusters showed significant differences among most of the sagittal parameters. The results showed that the second cluster has less severe retrognathism of the mandible, which is represented by a lower ANB angle and Calculated_ANB and a higher SNB angle. Interestingly, age differences were significant between clusters, among males and females in SCIIMO patients. These results can have implications both on the diagnosis and treatment plan

The ML models showed a high ability to predict a SCIO or SCIIMO with an accuracy of 0.87 (RF, accuracy = 0.87, kappa = 0.74, ROC = 0.92, sensitivity = 0.92, and specificity = 0.83) in the general model and a 0.78 accuracy (KNN, accuracy = 0.78, kappa = 0.56, ROC = 0.82, sensitivity = 0.80, and specificity = 0.76) using Wits appraisal and ANB angle. The study presents a machine learning model as a promising universal approach for precise and fast skeletal class I/ II diagnosis, advancing personalized orthodontic diagnostics and treatment. Finally, further research is recommended to be conducted on applying artificial intelligence and machine learning methods on the treatment choices and outcomes.

## Figures and Tables

**Table 1:** Descriptive statistics for skeletal class I patients—detailed information about skeletal class I patients. (N)—sample size, (M)—mean, (Std. Dev.)—standard deviation, (Min)—minimum value, (Pctl. 25)—25th percentile, (Pctl. 75)—75th percentile, (Max)—maximum value.

	Class I						
Variable	N	Mean	Std. Dev.	Min	Pctl. 25	Pctl. 75	Max
Age	157	19	7.1	9	14	21	55
0 < Age < 13	30 (19%)						
14 < Age < 20	85 (54%)						
Age > 21	42 (27%)						
Female	104 (66%)						
Male	53 (34%)						
NL-ML angle	157	30	6	13	26	34	46
NL-NSL angle	157	7.7	3.3	0.4	5.2	9.9	16
PFH/AFH	157	65	5	50	62	68	79
Gonial angle	157	133	7.4	113	128	138	155
Facial axis	157	88	4.5	75	85	91	103
SNA angle	157	83	3.9	75	80	85	94
SNB angle	157	78	3.6	68	76	80	86
ANB angle	157	4.6	1.9	0.3	3.2	5.8	9
ANB_ind_	157	5.4	1.4	2.2	4.5	6.3	8.8
Calculated_ANB (ANB − ANB_ind_)	157	−0.81	1.3	−4.6	−1	−0.1	2.3
SN-Ba angle	157	129	5.8	113	125	132	141
SN-Pg angle	157	79	3.8	67	76	81	89
S-N (mm)	157	63	7.5	39	58	65	88
Go-Me (mm)	157	60	6.6	46	55	63	82
Wits appraisal (mm)	157	1.7	2.5	−6.4	0.2	3.1	8.3
ML-NSL angle	157	37	6.9	7.5	34	41	61
+1/NL angle	157	114	5.9	97	110	119	128
+1/SNL angle	156	107	7.2	84	101	111	125
+1/NA angle	157	24	6.3	2.8	20	29	40
+1/NA (mm)	157	3.7	2.2	−2.3	2.2	5.2	10
−1/ML (anatomic)	157	93	7.3	72	88	97	111
−1/NB angle	157	28	6.5	10	24	32	47
−1/NB (mm)	157	5.5	2.3	−1.4	3.8	6.9	12
Interincisal angle	157	124	10	102	118	131	154

**Table 2:** Descriptive statistics for Skeletal class II patients—detailed information about tested skeletal class II patients. (N)—sample size, (M)—mean, (Std. Dev.)—standard deviation, (Min)—minimum value, (Pctl. 25)—25th percentile, (Pctl. 75)—75th percentile, (Max)—maximum value.

	Class II						
Variable	N	Mean	Std. Dev.	Min	Pctl. 25	Pctl. 75	Max
Age	237	17	6.5	7	13	20	43
0 < Age < 13	73 (31%)						
14 < Age < 20	110 (46%)						
Age > 21	54 (23%)						
Female	158 (67%)						
Male	79 (33%)						
NL-ML angle	237	29	6.5	8.6	24	34	54
NL-NSL angle	237	8.1	3.7	1.8	5.8	9.8	42
PFH/AFH	237	65	5.1	50	62	68	82
Gonial angle	237	129	7.4	110	124	134	150
Facial axis	237	88	4.5	70	85	91	102
SNA angle	237	83	3.7	74	80	85	93
SNB angle	237	76	5.5	7.8	74	78	84
ANB angle	237	6.8	2.3	−1.3	5.4	8.3	16
ANB_ind_	237	5.3	1.6	1.5	4.2	6.4	10
Calculated_ANB (ANB − ANB_ind_)	237	1.5	1.5	−4.9	1.1	2.2	5.5
SN-Ba angle	237	130	5.4	118	126	133	147
SN-Pg angle	237	77	3.6	65	74	79	86
S-N (mm)	237	63	7.2	30	59	64	87
Go-Me (mm)	237	59	5.9	46	55	62	79
Wits appraisal (mm)	237	4.6	3.3	−4.4	2.3	6.8	13
ML-NSL angle	237	37	6.7	16	33	41	63
+1/NL angle	237	113	7.5	91	108	118	135
+1/SNL angle	237	105	8	78	100	110	125
+1/NA angle	237	23	7.9	−2.4	17	28	42
+1/NA (mm)	237	3.1	2.4	−2.8	1.3	4.7	9.2
−1/ML (anatomic)	236	95	7.7	75	91	101	120
−1/NB angle	237	28	7.3	3.4	24	33	50
−1/NB (mm)	237	5.5	2.5	−0.1	3.6	7.4	12
Interincisal angle	237	123	11	100	115	130	158

**Table 3:** Hierarchical clustering results summary according to their skeletal classification. Summary of hierarchical Ward clustering results when using all variables. Represents number of patients in each cluster and their classification (separate clustering for each skeletal classification class I and II).

Parameters Included	Cluster		Class Calculated ANB	Class Calculated ANB
			I	II
All	1		75	125
	2		54	62
	3		27	49
Total			156	236
Males	1		20	20
	2		18	27
	3		15	31
Total			53	78
Females		1	25	58
		2	50	75
		3	28	25
Total			103	158

**Table 4:** Hierarchical clustering analysis for skeletal class I patients. Cephalometric parameters, descriptive statistics, mean, and standard deviation (Std. Dev.) for each cluster, for skeletal class I patients. In addition, table presents ANOVA significance levels of comparisons between the three clusters in each malocclusion group (NS—not significant, * <0.05, ** <0.01, and *** <0.001).

Parameter	Class I Malocclusion						
	Cluster 1		Cluster 2		Cluster 3		
	Mean	Std. Dev.	Mean	Std. Dev.	Mean	Std. Dev.	Sig ANOVA
Age	17.95	4.84	19.73	9.01	18.89	7.30	NS
NL-ML angle [°]	32.27	6.07	29.21	4.13	24.66	5.35	***
NL-NSL angle [°]	8.27	3.54	8.32	2.68	5.21	2.70	***
PFH/AFH (%)	62.93	4.85	64.54	3.40	70.32	4.05	***
Gonial angle [°]	135.33	7.82	131.86	4.64	127.57	7.59	***
Facial axis	86.62	4.17	88.42	3.88	91.86	4.40	***
SNA angle [°]	82.29	3.67	80.89	2.98	86.64	3.42	***
SNB angle [°]	76.92	3.25	77.56	3.30	81.66	2.73	***
ANB angle [°]	5.37	1.62	3.32	1.36	4.97	2.05	**
ANB_ind_ [°]	5.86	1.48	4.73	0.97	5.45	1.60	*
Calculated_ANB (ANB − ANB_ind_) [°]	−0.49	1.01	−1.41	1.49	−0.48	1.30	NS
SN-Ba angle [°]	129.47	5.95	130.24	4.95	124.69	5.24	**
SN-Pg angle [°]	77.65	3.60	78.14	3.45	82.34	2.97	***
S-N (mm)	60.75	7.63	63.05	6.31	66.74	8.01	***
Go-Me (mm)	57.83	6.59	60.18	5.54	62.96	7.00	***
Wits appraisal (mm)	1.62	2.27	0.88	2.69	3.25	1.97	NS
ML-NSL angle [°]	40.22	6.29	37.29	4.16	29.28	6.60	***
+1/NL angle [°]	110.76	5.20	116.63	4.08	119.09	4.85	***
+1/SNL angle [°]	102.44	6.59	108.41	4.61	113.99	5.60	***
+1/NA angle [°]	20.29	5.44	27.33	4.23	27.69	6.17	***
+1/NA (mm)	2.56	1.63	4.64	1.87	5.21	2.57	***
−1/ML (anatomic)	89.76	6.53	92.69	6.23	100.18	5.87	***
−1/NB angle [°]	26.71	6.86	27.66	5.68	31.70	5.62	**
−1/NB (mm)	5.35	2.32	5.07	2.06	6.75	2.52	*
Interincisal angle [°]	128.52	10.42	121.75	7.37	116.04	6.66	***

**Table 5:** Hierarchical clustering analysis for skeletal class I patients with gender effect. Cephalometric parameters, descriptive statistics, mean, and standard deviation (Std. Dev.) for each cluster, including skeletal class I, but with each gender separately. In addition, table presents ANOVA significance levels of comparisons between the three clusters in each malocclusion group (NS—not significant, * <0.05, ** <0.01, and *** <0.001).

	Class I Males							Class I Females						
Cluster	1		2		3		Sig ANOVA	1		2		3		Sig ANOVA
	Mean	Std. Dev.	Mean	Std. Dev.	Mean	Std. Dev.		Mean	Std. Dev.	Mean	Std. Dev.	Mean	Std. Dev.	
Age	16.01	4.04	17.73	6.48	17.59	3.11	NS	16.63	4.76	21.22	9.48	19.35	5.45	NS
NL-ML angle [°]	31.72	5.40	25.45	5.85	30.57	5.74	NS	36.34	4.66	27.65	4.41	29.34	5.32	***
NL-NSL angle [°]	8.72	3.23	5.11	3.23	6.55	3.08	*	7.79	3.72	9.09	2.76	7.02	2.88	NS
PFH/AFH (%)	63.58	4.11	69.72	4.73	65.69	3.31	NS	60.66	5.77	64.91	3.50	65.34	5.08	***
Gonial angle [°]	135.85	6.95	128.03	7.59	132.75	6.86	NS	139.99	5.59	130.45	6.37	131.40	5.84	***
Facial axis	87.17	4.80	91.83	4.87	86.88	4.91	NS	84.83	4.01	88.89	2.94	88.82	4.50	***
SNA angle [°]	79.68	3.28	85.22	3.50	85.58	3.06	***	82.49	3.31	81.23	3.61	83.72	3.73	NS
SNB angle [°]	75.77	2.95	81.39	3.02	78.99	2.44	**	76.61	3.81	77.41	3.40	78.98	3.20	*
ANB angle [°]	3.94	1.30	3.83	1.91	6.59	1.30	***	5.89	1.30	3.82	1.59	4.70	1.96	*
ANB_ind_ [°]	4.79	1.40	5.01	1.63	6.50	1.21	**	6.64	0.65	4.67	1.19	5.67	1.22	*
Calculated_ANB (ANB − ANB_ind_) [°]	−0.86	1.05	−1.18	1.70	0.09	0.79	NS	−0.75	1.03	−0.86	1.53	−0.97	1.12	NS
SN-Ba angle [°]	130.94	5.10	125.87	6.01	124.83	5.79	**	129.02	5.40	131.29	5.73	127.26	4.25	NS
SN-Pg angle [°]	76.67	2.95	82.08	3.52	79.59	3.04	*	76.90	4.29	78.14	3.40	79.74	3.54	**
S-N (mm)	60.52	8.82	68.34	9.05	67.02	8.96	*	57.64	3.56	62.10	5.81	63.25	6.80	***
Go-Me (mm)	58.27	5.13	63.22	7.85	62.13	9.72	NS	54.66	3.79	59.65	5.14	60.79	6.54	***
Wits appraisal (mm)	0.62	2.73	2.90	2.50	2.92	1.61	**	1.36	2.53	1.28	2.59	1.79	2.07	NS
ML-NSL angle [°]	39.56	6.06	30.46	5.31	37.13	4.92	NS	43.87	6.73	36.52	4.08	35.75	8.19	***
+1/NL angle [°]	114.31	3.92	119.83	3.82	107.61	6.05	**	110.62	4.55	113.14	4.41	119.31	4.35	***
+1/SNL angle [°]	105.69	5.21	114.90	4.58	101.19	7.32	NS	102.84	6.67	103.77	4.96	112.69	5.17	***
+1/NA angle [°]	25.91	3.91	29.68	3.83	15.52	5.85	***	20.56	4.59	22.68	4.78	29.02	4.93	***
+1/NA (mm)	4.02	1.73	5.74	2.14	1.09	1.67	**	2.81	1.21	3.20	1.55	5.47	2.22	***
−1/ML (anatomic)	90.16	6.97	97.39	8.07	92.79	6.14	NS	87.31	5.94	91.36	5.89	97.97	6.22	***
−1/NB angle [°]	25.94	7.14	29.34	6.60	28.73	6.97	NS	28.05	6.08	25.15	4.52	32.71	6.34	**
−1/NB (mm)	4.93	2.32	6.23	2.89	5.46	2.38	NS	6.09	1.50	4.45	1.89	6.77	2.46	NS
Interincisal angle [°]	125.58	10.60	117.24	8.38	130.14	12.03	NS	126.23	6.98	128.44	7.47	114.08	6.15	***

**Table 6:** Hierarchical clustering analysis for skeletal class II patients. Cephalometric parameters, descriptive statistics, mean, and standard deviation (Std. Dev.) for each cluster, when including skeletal class II. In addition, table presents ANOVA significance levels of comparisons between the three clusters in each malocclusion group (NS—not significant, * <0.05, ** <0.01, and *** <0.001).

Parameter	Class II Malocclusion						
	Cluster 1		Cluster 2		Cluster 3		
	Mean	Std. Dev.	Mean	Std. Dev.	Mean	Std. Dev.	Sig ANOVA
Age	17.59	7.20	15.50	3.83	18.20	7.04	NS
NL-ML angle [°]	30.25	5.68	24.15	4.91	31.49	7.13	NS
NL-NSL angle [°]	7.82	4.16	7.89	2.83	9.15	3.40	NS
PFH/AFH (%)	64.01	4.54	68.18	4.58	62.56	4.88	NS
Gonial angle [°]	130.62	7.29	124.60	6.44	131.42	6.27	NS
Facial axis	87.95	3.98	90.22	3.77	84.13	4.57	***
SNA angle [°]	83.42	3.56	82.12	4.05	81.67	3.42	**
SNB angle [°]	76.21	2.85	77.22	3.34	72.21	9.84	***
ANB angle [°]	7.17	1.93	4.90	2.02	8.16	1.94	NS
ANB_ind_ [°]	5.76	1.45	4.13	1.25	5.63	1.73	*
Calculated_ANB (ANB − ANB_ind_) [°]	1.40	1.34	0.77	1.63	2.53	1.15	***
SN-Ba angle [°]	129.79	5.63	129.89	5.04	130.18	5.22	NS
SN-Pg angle [°]	76.95	2.95	78.72	3.28	73.65	3.24	***
S-N (mm)	64.09	7.80	60.68	6.39	61.58	5.89	**
Go-Me (mm)	59.64	6.51	59.03	4.35	55.87	5.10	***
Wits appraisal (mm)	5.09	3.44	3.09	2.90	5.46	3.06	NS
ML-NSL angle [°]	37.91	5.72	32.01	5.45	40.65	6.74	NS
+1/NL angle [°]	117.33	5.76	109.92	5.38	105.64	5.90	***
+1/SNL angle [°]	109.91	5.87	102.15	5.63	96.49	6.25	***
+1/NA angle [°]	26.65	6.04	20.34	6.81	14.86	6.46	***
+1/NA (mm)	4.35	2.09	2.05	1.96	1.21	1.73	***
−1/ML (anatomic)	96.30	7.24	92.48	8.35	96.69	7.03	NS
−1/NB angle [°]	30.61	5.64	21.99	7.29	30.94	6.45	NS
−1/NB (mm)	6.43	2.14	3.14	1.64	6.08	2.54	**
Interincisal angle [°]	116.10	7.28	133.34	8.85	126.15	8.73	***

**Table 7:** Hierarchical clustering analysis for skeletal class II patients with gender effect. Cephalometric parameters, descriptive statistics, mean, and standard deviation (Std. Dev.) for each cluster, including the skeletal class II patients, but with each gender separately. In addition, table presents ANOVA significance levels of comparisons between the three clusters in each malocclusion group (NS—not significant, * < 0.05, ** < 0.01, and *** < 0.001).

	Class II Males							Class II Females						
Cluster	1		2		3		Sig ANOVA	1		2		3		Sig ANOVA
	Mean	Std. Dev.	Mean	Std. Dev.	Mean	Std. Dev.		Mean	Std. Dev.	Mean	Std. Dev.	Mean	Std. Dev.	
Age	12.90	2.20	17.58	7.09	16.98	6.39	*	15.88	5.11	18.76	7.71	18.56	5.53	*
NL-ML angle [°]	30.66	4.50	23.91	5.06	31.93	6.56	NS	27.73	4.80	32.35	5.73	21.59	4.34	*
NL-NSL angle [°]	7.78	3.04	6.67	3.04	8.16	3.40	NS	9.28	2.50	8.50	4.89	6.03	2.21	**
PFH/AFH (%)	62.79	2.75	70.12	3.29	62.72	3.62	NS	64.62	3.60	62.03	4.00	72.00	4.18	***
Gonial angle [°]	131.55	5.80	125.35	8.20	131.92	6.07	NS	128.24	6.75	132.11	6.44	121.64	5.80	*
Facial axis	89.40	3.37	90.53	3.88	85.44	4.43	***	87.27	3.20	86.29	4.83	91.78	3.78	**
SNA angle [°]	79.16	2.73	84.61	3.45	83.37	3.23	***	80.69	2.58	83.19	3.72	85.99	2.99	***
SNB angle [°]	71.62	15.21	78.30	2.19	75.17	3.15	NS	74.81	2.53	75.18	3.18	79.93	2.38	***
ANB angle [°]	4.35	2.10	6.33	1.94	8.19	1.46	***	5.87	1.92	7.95	1.98	6.03	2.28	NS
ANB_ind_ [°]	4.19	1.28	4.81	1.83	6.21	1.50	***	4.54	1.24	6.18	1.42	4.75	1.29	*
Calculated_ANB (ANB − ANB_ind_) [°]	0.16	1.86	1.51	0.90	1.98	1.11	***	1.32	1.56	1.78	1.46	1.28	1.61	NS
SN-Ba angle [°]	132.49	5.93	128.94	3.43	129.23	5.55	*	130.73	5.69	129.79	5.57	128.06	4.36	*
SN-Pg angle [°]	75.47	2.17	79.43	2.29	75.35	3.55	NS	75.93	2.71	75.83	3.50	81.08	2.64	***
S-N (mm)	65.31	8.55	62.81	6.49	66.27	7.58	NS	60.15	3.80	63.19	7.69	60.27	8.57	NS
Go-Me (mm)	59.86	8.26	58.31	6.03	61.29	6.31	NS	56.70	4.39	58.63	6.16	59.82	3.67	**
Wits appraisal (mm)	2.84	2.96	4.81	2.67	5.34	3.32	**	3.68	3.47	5.48	3.28	4.75	3.45	*
ML-NSL angle [°]	38.49	3.76	30.51	4.27	40.09	5.66	NS	37.06	4.77	40.43	5.74	27.89	4.50	***
+1/NL angle [°]	113.42	7.25	114.98	6.84	109.57	8.16	*	108.31	5.22	115.62	6.25	117.43	8.46	***
+1/SNL angle [°]	105.70	6.99	108.34	7.63	101.74	9.00	NS	99.11	5.63	107.67	6.27	111.38	7.50	***
+1/NA angle [°]	26.79	7.19	23.70	6.46	18.08	7.53	***	18.87	6.56	24.65	7.44	25.60	9.08	***
+1/NA (mm)	4.94	2.59	3.00	2.31	2.35	2.31	***	1.74	1.72	3.81	2.28	3.65	2.42	***
−1/ML (anatomic)	90.43	4.57	98.56	6.57	98.90	8.38	***	91.25	6.76	95.56	6.81	100.52	7.58	***
−1/NB angle [°]	23.85	4.22	27.64	8.33	34.23	6.57	***	23.34	6.38	31.40	5.14	28.54	7.39	***
−1/NB (mm)	4.25	2.07	4.73	1.98	7.80	2.13	***	3.53	1.56	6.86	2.10	4.92	2.42	***
Interincisal angle [°]	125.30	7.49	122.58	12.99	119.85	8.68	NS	132.57	8.35	116.48	7.73	120.19	10.89	***

**Table 8:** Stepwise Forward Machine Learning Models, including General Model, Model 1, Model 2, and Model 3: These rows represent different models used for prediction, potentially containing various combinations of cephalometric parameters. General Model included all parameters. In Models 1-3, sign (−) indicates that parameter was not included, while (+) indicates that parameter was included.

	ANB	Wits Appraisal	−1/NB Angle	Gonial Angle	Best Model	Accuracy	Kappa	ROC Curve	Sensitivity	Specificity
General model					RF, CART	0.87	0.74	RF = 0.92, CART = 0.90	RF = 0.92, CART = 0.94	RF = 0.83, CART = 0.82
Model 1	(+)	(−)	(−)	(−)	LDA	0.75	0.47	0.79	0.59	0.86
Model 2	(+)	(+)	(−)	(−)	KNN	0.78	0.56	0.82	0.80	0.76
Model 3	All variables except ANB, ANB_ind_, and Calculated_ANB				LDA	0.82	0.63	0.88	0.75	0.87

## Data Availability

The data supporting this study’s findings are available on request from the corresponding author.

## Supplementary Materials

The following supporting information can be downloaded at: https://www.mdpi.com/article/10.3390/jcm14030792/s1. Table S1. Cephalometric parameters definitions. Figure S1. Lateral cephalogram parameters.

## Abbreviations

SCIO	skeletal class I occlusion
SCIIMO	skeletal class II malocclusion
ML	machine learning
3D	three-dimensional
SVM	Support Vector Machine
CNN	convolutional neural network
LDA	Linear Discriminant Analysis
KNN	K-Nearest Neighbor
RF	Random Forest
CART	Classification and Regression Tree
ROC	receiver–operating characteristic curve
Descriptive statistics tables:	
(N)	sample size
(M)	mean
(SD/Std. Dev.)	standard deviation
(Min)	minimum value
(Pctl. 25)	25th percentile
(Pctl. 75)	75th percentile
(Max)	maximum value
NS	not significant
ANB_ind_	ANB individual
